# A mitochondria-targeted nanoradiosensitizer activating reactive oxygen species burst for enhanced radiation therapy†
†Electronic supplementary information (ESI) available. See DOI: 10.1039/c7sc04458e


**DOI:** 10.1039/c7sc04458e

**Published:** 2018-02-28

**Authors:** Na Li, Longhai Yu, Jianbo Wang, Xiaonan Gao, Yuanyuan Chen, Wei Pan, Bo Tang

**Affiliations:** a College of Chemistry , Chemical Engineering and Materials Science , Collaborative Innovation Center of Functionalized Probes for Chemical Imaging in Universities of Shandong , Key Laboratory of Molecular and Nano Probes , Ministry of Education , Institute of Molecular and Nano Science , Shandong Normal University , Jinan 250014 , P. R. China . Email: tangb@sdnu.edu.cn; b Radiation Department , Qilu Hospital of Shandong University , Jinan 250100 , P. R. China

## Abstract

Radiation therapy (RT) has been widely used for malignant tumor treatment. However, the large dosage of ionizing radiation and high frequency of radiotherapy in clinical cancer therapy cause severe damage to normal tissues adjacent to tumors. Therefore, how to increase the local treatment efficacy and reduce the damage to normal tissues has been a challenge for RT. Herein, we developed a novel strategy for enhanced RT based on a mitochondria targeted titanium dioxide-gold nanoradiosensitizer. When irradiated with X-rays, the nanosensitizer could produce reactive oxygen species (ROS) in the mitochondria, which induced the domino effect on the ROS burst. The overproduced ROS accumulated in mitochondria, resulting in mitochondrial collapse and irreversible cell apoptosis. A colony formation assay indicated that the cell survival rate when incubated with the mitochondrial targeted nanosensitizer was significantly lower than that of non-targeted groups. As demonstrated by *in vivo* experiments, the tumor was significantly suppressed even just once RT with the nanosensitizer.

## Abstract 2

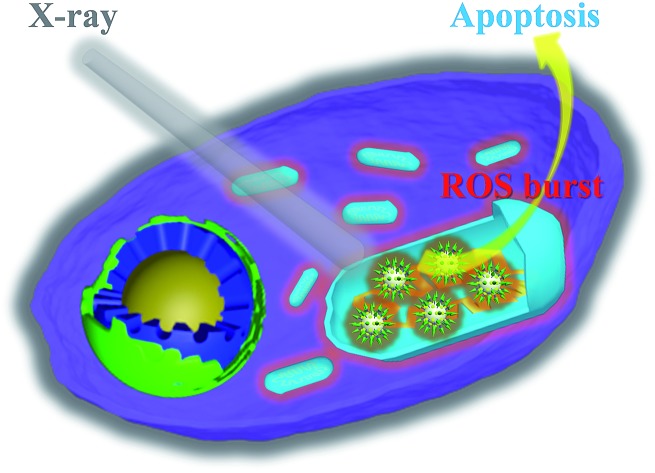
We developed a novel strategy for enhanced radiation therapy based on a mitochondria targeted titanium dioxide-gold nanoradiosensitizer.

## Introduction

Malignant tumors have been a serious threat to human health with their increasing incidence. Radiation therapy (RT), as one of the most common cancer treatments, draws great attention in the medical field.^1^–^3^ As radiation does not have the ability to target tumor cells, and because of the large dosage and multiple rounds of radiation needed during the course of treatment, a large number of normal cells are killed when destroying tumor cells, which leads to serious side effects as well. Recently, many radiosensitizers have been developed to selectively improve the sensitivity of tumor tissues towards high energy radiation^4^,^5^ and reactive oxygen species (ROS) were mainly generated in the cytoplasm of tumor cells, which limited the enhancement of the therapeutic effects. Moreover, the produced ROS have extremely short lifespans and a limited diffusion distance,^6^–^10^ leading to the low killing efficiency of tumor cells and unsatisfactory therapeutic effects.^11^–^13^ Therefore, it is necessary to develop a more effective method to improve the therapeutic effect of RT, which could decrease the number of doses of radiation and increase the killing efficiency of ROS for tumor tissues.

Mitochondria are the primary organelles that produce up to ninety percent of ROS in living cells.^14^,^15^ The imbalance of ROS in mitochondria can cause mitochondrial dysfunction, which is the decisive factor in the pathways of cell apoptosis.^16^–^18^ This is considered to be a dominant mode of apoptosis in cancer treatment, which indicates that mitochondria play a key role in radiotherapy sensitization.^19^–^21^ If the RT sensitizer is localized in the mitochondria, the generated ROS can accumulate in the mitochondria and will lead to mitochondrial dysfunction, thereby enabling the continuous production of ROS. Such initiation of the domino effect on ROS burst could effectively destroy cancer cells and greatly decrease the dosage and frequency of radiation, as well as increase the therapeutic efficiency of RT.^22^

**Scheme 1:**
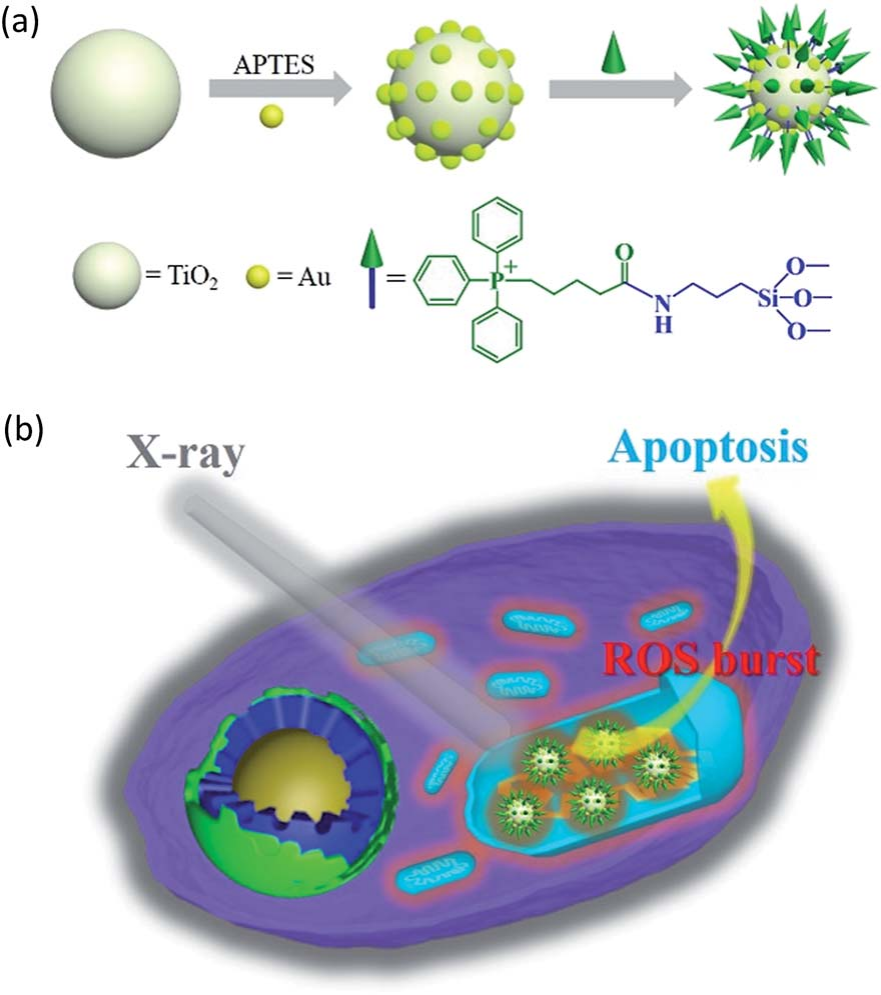
Schematic illustration of the synthesis of the TiO_2_–Au–TPP NPs (a) and radiation therapy in the cell’s mitochondria with nanoradiosensitizers (b).

In this study, we constructed an X-ray responsive nanosensitizer to induce the domino effect on mitochondrial ROS burst. The nanosensitizer, based on titanium dioxide nanoparticles (TiO_2_ NPs), is modified with gold nanoparticles (Au NPs) to build a satellite structure. Triphenylphosphine (TPP), as a mitochondria-targeted group, was covalently attached to the surface of TiO_2_ to achieve the mitochondrial targeting effect. The nanoradiosensitizer could selectively trigger the localized ROS outburst in mitochondria under irradiation with X-rays, which resulted in mitochondrial collapse and irreversible cell apoptosis by accumulation of the overproduced ROS in mitochondria. The nanoradiosensitizer not only reduces the amount and frequency of radiation, but also shortens the treatment cycle in RT. The details of this process are presented in Scheme 1.

## Results and discussion

### Synthesis and characterization of the nanosensitizers

**Fig. 1:**
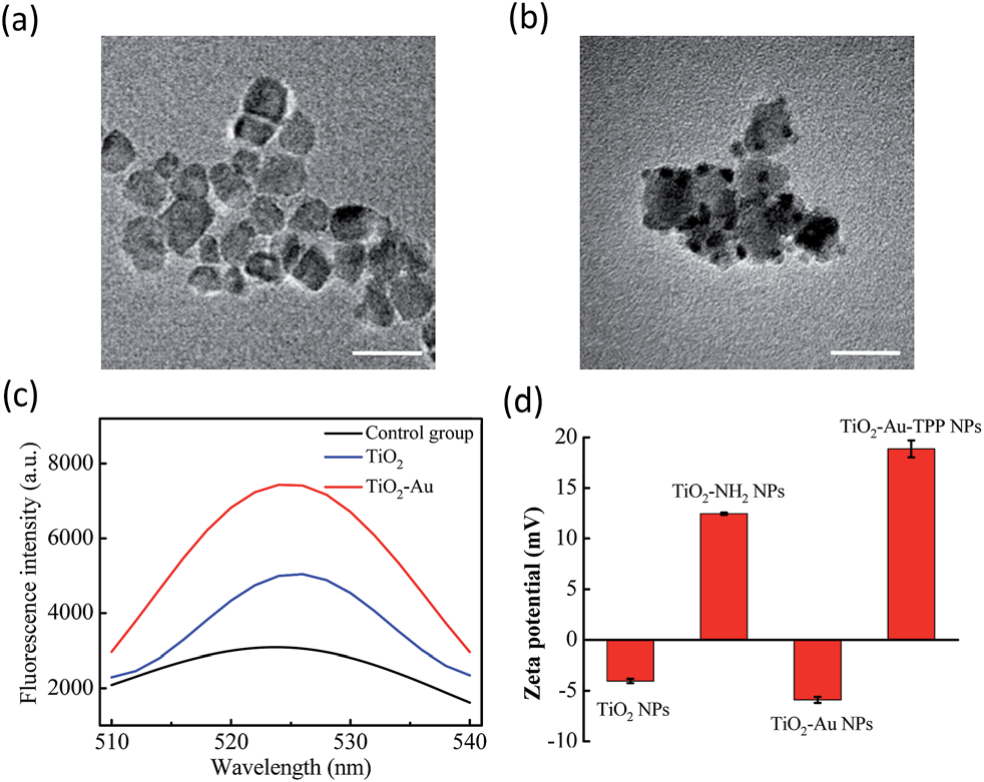
Characterization of the nanoradiosensitizers. HRTEM images of the (a) TiO_2_ NPs and (b) TiO_2_–Au NPs; the scale bars are 20 nm. (c) Fluorescence spectra of DBZTC (excitation = 490 nm, emission = 525 nm) after irradiation with X-rays. (d) Zeta potentials of each step of the modification: TiO_2_ NPs; TiO_2_–NH_2_ NPs; TiO_2_–Au NPs; and TiO_2_–Au–TPP NPs.

The TiO_2_ NPs were prepared *via* a previously reported sol–gel method with further modification.^23^ The high resolution transmission electron microscopy (HRTEM) images display the NPs’ fine size control for an average diameter of ∼18 nm with excellent dispersity (Fig. 1a). With further decoration with 3–5 nm Au NPs, the satellite structure can be used as a nanosensitizer (Fig. 1b). The crystal structure of the NPs was investigated by powder X-ray diffraction (PXRD), which showed that the crystalline form of synthetic TiO_2_ was anatase (Fig. S1, ESI†). Zeta potential studies of the surface charge of the NPs were performed to demonstrate the process of attaching a functional group. The zeta potential of TiO_2_ is at –4.1 ± 0.2 mV, which represents the negatively charged hydroxyl group on the surface, and changes to 12.5 ± 0.1 mV after functionalization with –NH_2_. As the Au NPs are negatively charged, the electron pair on the TiO_2_ coordinates with the empty orbital of the Au NPs, leading to a decrease of the zeta potential to –5.9 ± 0.3 mV. Finally, the attachment of TPP onto the TiO_2_–Au NPs again raises the potential to 18.9 ± 0.8 mV, demonstrating the successful preparation of the nanosensitizer TiO_2_–Au–TPP (Fig. 1d).

In order to test the superoxide anion (O_2_˙^–^) productivity of the nanosensitizer, a special nanoprobe 2-chloro-1,3-dibenzothiazoline-cyclohexene (DBZTC) was used for the detection of O_2_˙^–^.^24^ After 4 Gy X-ray irradiation on both the TiO_2_ NPs and TiO_2_–Au NPs, the TiO_2_–Au NPs exhibit significantly enhanced fluorescence emission signals at 525 nm, indicating more effective production of O_2_˙^–^ (Fig. 1c). Furthermore, the amount of O_2_˙^–^ produced from the TiO_2_–Au NPs upon X-ray irradiation was quantified as 0.174 μmol per mg using fluorescence spectra (Fig. S3, ESI†). The results indicated that the nanosensitizer can effectively produce O_2_˙^–^.

### Triphenylphosphine (TPP) concentration optimization

The amount of TPP on the nanoradiosensitizer can affect the targeting ability for mitochondria, which further affects the ROS burst. The concentration of the TPP group was optimized through the colocalization ability of the nanosensitizer. Typically, nanoparticles (TiO_2_–Au–TPP-IR806) with a series of TPP concentrations were prepared, and then incubated with the MCF-7 cells. Mitochondria were labelled with MTG before the imaging experiment (Fig. S4, ESI†). The colocalization effect was significantly enhanced until the TPP loading capacity went up to 16 μmol (the TPP concentration was calculated to be 3.83 μmol mg^–1^ TiO_2_–Au using UV-vis spectra, Fig. S2, ESI†), and then reached a plateau with no obvious change, indicating the best colocalization effect of TPP with the Pearson’s correlation coefficient reaching the maximum value (*ρ* = 0.643). Furthermore, the cell survival rate reduced to below 30% when the amount of TPP added was 16 μmol (Fig. S5, ESI†), thereby the TPP loading capacity of 16 μmol was chosen in the following steps. The results indicate that the nanoradiosensitizer can specifically target mitochondria for future cancer therapy applications.

### Real-time monitoring of intracellular O_2_˙^–^ burst

**Fig. 2:**
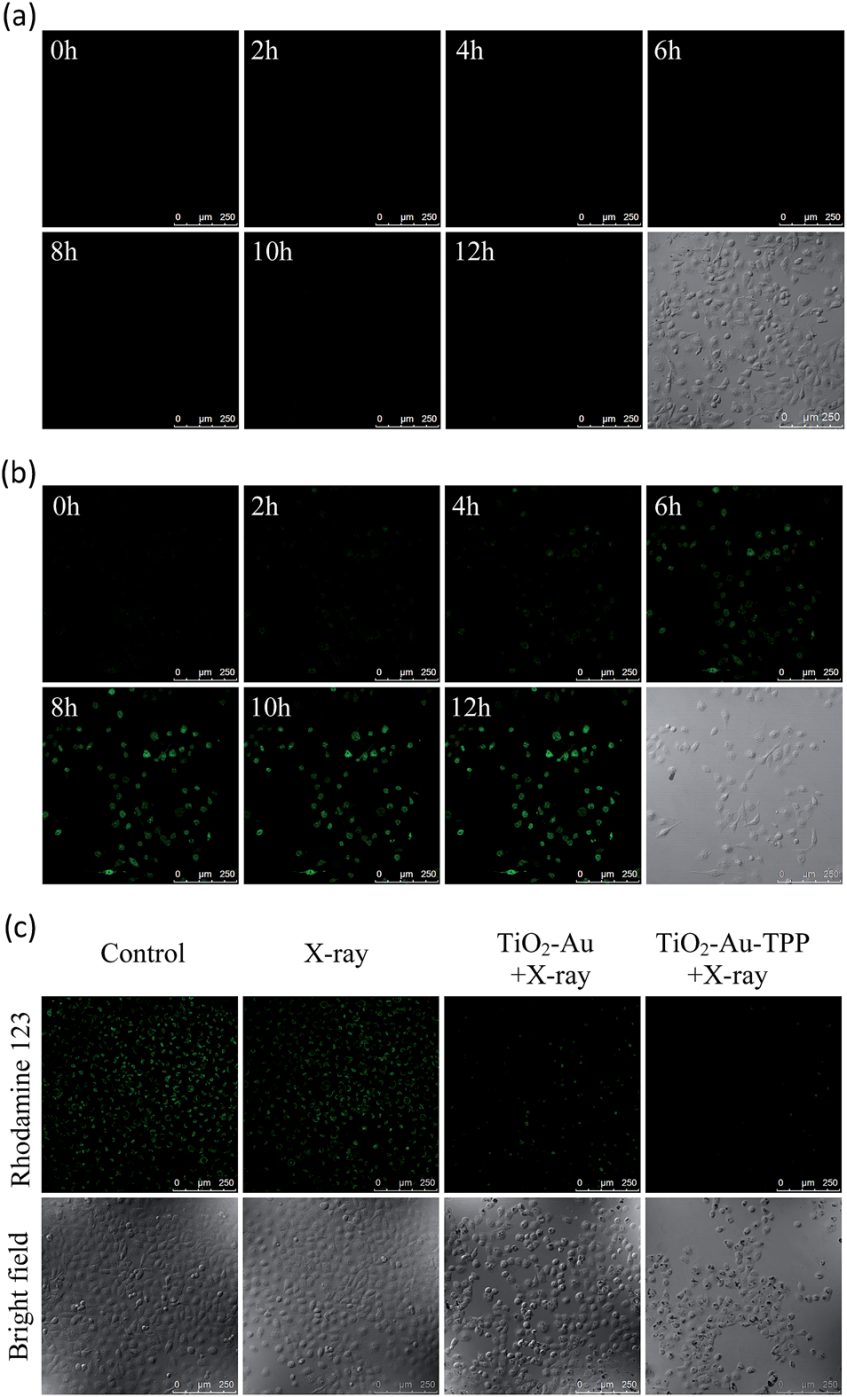
Real-time imaging of intracellular O_2_˙^–^ burst and detection of the mitochondrial membrane potential (Δ*ψ*_m_). The MCF-7 cells incubated with (a) TiO_2_–Au–HE or (b) TiO_2_–Au–TPP–HE 12 h post irradiation with X-rays and images were obtained at 2 h intervals. Confocal images of the four groups of MCF-7 cells were taken after different treatments, then the MCF-7 cells were incubated with Rhodamine 123. Confocal images of Rhodamine 123-stained cells (c) are obtained.

Since hydroethidine (HE) is a commonly used fluorescent probe for the detection and real-time monitoring of O_2_˙^–^, the TiO_2_–Au–TPP–HE nanoprobe was applied to investigate its induction of the domino activation for ROS in mitochondria. After incubation of the MCF-7 cells with TiO_2_–Au–HE and TiO_2_–Au–TPP–HE, respectively, 4 Gy X-rays were used to irradiate cells. The fluorescence intensity of TiO_2_–Au–TPP–HE in MCF-7 cells was monitored by confocal laser scanning microscopy (CLSM). Cells with TiO_2_–Au–HE treatment display weak fluorescence intensity for 12 h, which represents a low production of ROS (Fig. 2a). However, cells with TiO_2_–Au–TPP–HE treatment exhibit enhanced fluorescence with time, which can be attributed to the continuous domino burst activation of the ROS (Fig. 2b). Moreover, the X-rays’ effect on ROS expression in tumor cells was further confirmed by a significant cell morphology change 12 h after irradiation, resulting in tumor cells’ apoptosis with the domino effect.^25^,^26^ In order to intuitively demonstrate the fluorescence intensity contrast, both fluorescence intensities were quantified (Fig. S6a, ESI†). The fluorescence intensity of the TiO_2_–Au–HE group is quite weak, but that of the TiO_2_–Au–TPP–HE group displays a strong signal and, in addition, the intensity increases with time.

### Determination of mitochondrial membrane potential (Δ*ψ*_m_)

It has been reported that intracellular mitochondrial membrane potential reduction occurs early in the process of cell apoptosis,^27^ and the changes can be detected by Rhodamine 123 staining.^28^,^29^ Hence, to study the influence of X-rays on the nanosensitizer towards the mitochondrial membrane potential, MCF-7 cells were incubated with TiO_2_–Au and TiO_2_–Au–TPP, respectively, for 8 h followed by 4 Gy X-ray irradiation. The fluorescence intensity of the samples with Rhodamine 123 was analysed by CLSM (Fig. 2c). The fluorescence intensity in the TiO_2_–Au group did not change significantly, while that of the TiO_2_–Au–TPP group dramatically weakened. The results demonstrate that TiO_2_–Au–TPP with X-rays can decrease the mitochondrial membrane potential, which was caused by the ROS activation of an inner membrane anion channel (IMAC) and acceleration of the mitochondrial permeability transition pore (MPTP) opening by oxidizing the matrix glutathione.^19^,^30^,^31^ The fluorescence intensity comparison was quantified and the ordinate was normalized to average the fluorescence intensity (Fig. S6b, ESI†). We also conducted a cell clone formation assay to verify the mechanism of mitochondrial membrane potential depolarization using cyclosporine A (CsA), a desensitizer of MPTP. As shown in Fig. S7,† the cell survival rate of the TiO_2_–Au–TPP with X-rays group was below 30% in the presence of CsA, which indicated that the opening of MPTP and activation of IMAC induce the decrease of Δ*ψ*_m_ synergistically.

### Caspase-3 activation

Caspase-3 activation is an important signal in ROS-mediated apoptotic signaling pathways. The activation of caspase-3 was verified using an immunofluorescence staining method. For the five MCF-7 cell groups with different treatments (control group, TiO_2_–Au–TPP only, X-rays only, TiO_2_–Au with X-rays, and TiO_2_–Au–TPP with X-rays), the fluorescence intensities of the immunofluorescence staining were measured. As shown in Fig. S8,† an increased activity of caspase-3 was observed when the MCF-7 cells were incubated with TiO_2_–Au–TPP for 8 h followed by 4 Gy X-ray irradiation. The results demonstrate that the nanosensitizer could activate caspase-3. Activated caspases can destroy the mitochondrial electron transport chain and result in the regeneration of ROS,^32^ and induce the domino effect on the ROS burst.

### Determination of mitochondrial oxygen consumption rate (OCR)

**Fig. 3:**
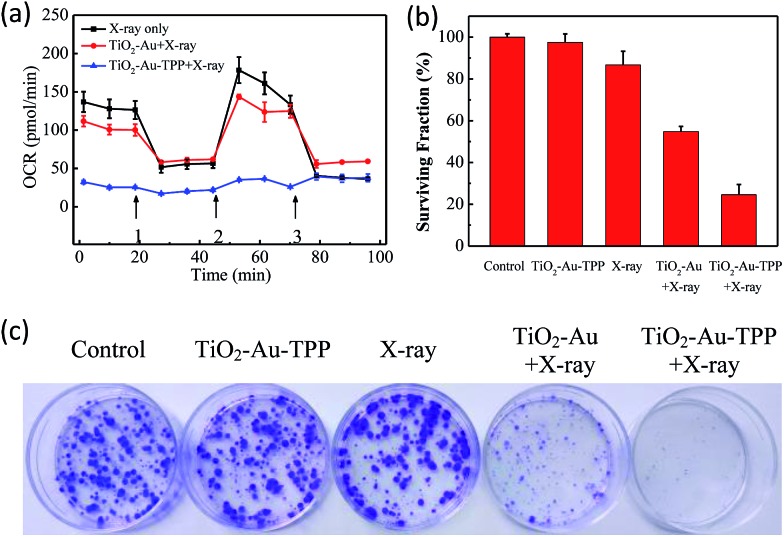
Determination of mitochondrial oxygen consumption rate and cell clone formation assay. Mitochondrial respiration profile of MCF-7 cells. Cells taken with different treatments and followed by real-time analysis of cellular respiration in XF24 cell culture plates (a) and surviving fractions of MCF-7 cells treated differently (b); photograph of the surviving fractions of MCF-7 cells after the different treatments (c).

The OCR values of intracellular mitochondria were measured to investigate the nanosensitizer’s function on the aerobic respiration of mitochondria.^33^ MCF-7 cells were firstly inoculated into XF24 cell culture plates and cultured for 24 h, then separately incubated with TiO_2_–Au and TiO_2_–Au–TPP for 8 h followed by 4 Gy X-ray irradiation. The cells were treated with a XF24 cell energy metabolism analyzer and the real-time monitoring of mitochondrial OCR values in different respiratory states was carried out. Oligomycin, as an ATP synthase inhibitor, reduces the oxygen consumption needed to assist ATP synthesis.^34^,^35^ Carbonyl cyanide 4-(trifluoromethoxy)phenylhydra-zone (FCCP), which behaves as an uncoupling agent for proton reflux, contributes a great amount of mitochondrial oxygen consumption, but not any ATP. Thus, the increase in oxygen consumption after the addition of FCCP represents the maximum oxygen consumption capacity of mitochondria.^36^ Rotenone, as the latest drug investigated, is an inhibitor of the mitochondrial aerobic respiratory chain, and completely inhibits mitochondrial oxygen consumption.^37^ The OCR values of the other two groups were significantly lower than that of the X-ray irradiation group, and the OCR of the TiO_2_–Au–TPP with X-rays group led to a rapid and pronounced reduction in basal and maximal mitochondrial oxygen consumption (Fig. 3a). As the results from the mitochondrial ROS burst and mitochondrial membrane potential reduction test coincide, together with X-ray irradiation TiO_2_–Au–TPP can cause intracellular ROS burst, leading to mitochondrial dysfunction and eventually cell apoptosis.

### Cell clone formation assay

In order to study the nanosensitizer’s inhibition performance towards tumor cells’ proliferation under X-ray irradiation, MCF-7 cells were evaluated using a cell clone formation trial.^38^ Through five different treatments on the cells (control group, TiO_2_–Au–TPP only, X-rays only, TiO_2_–Au with X-rays, and TiO_2_–Au–TPP with X-rays), the formed cell colonies were counted after 10 days (Fig. 3b and c). The number of colonies in the control group and TiO_2_–Au–TPP group is the largest, indicating that the nanosensitizer does not affect cell proliferation and exhibits outstanding biocompatibility. In addition, the X-ray group has a slightly lower number of cell colonies, which is consistent with the clinical use of X-ray RT on tumors. In this regard, to verify whether TiO_2_–Au–TPP is able to maximize the cell proliferation ability, the TiO_2_–Au with X-rays group was also used for comparison, and it displayed a significant decrease in the number of colonies. More importantly, the TiO_2_–Au–TPP with X-rays group produces the least number of colonies (the cell survival rate < 25%), since the proliferation of the tumor cells has been expressively inhibited by the nanosensitizers.

### Cell migration and invasion assays

**Fig. 4:**
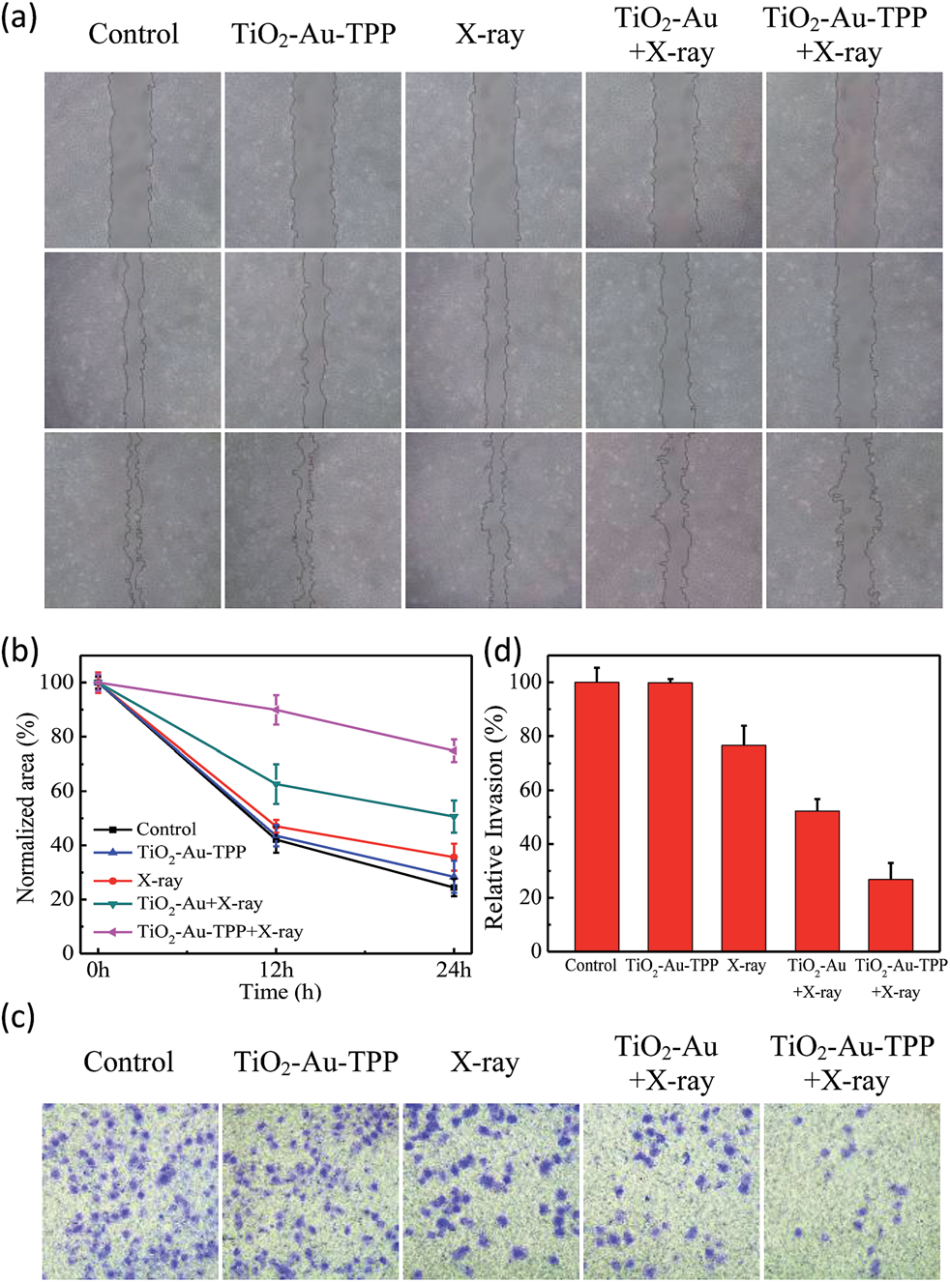
Wound-healing assay of cells treated with different treatments (a) at different time points after wounding; normalized area of empty area range with different treatments (b); a chamber invasion assay was conducted in the MCF-7 cells with different treatments. The invasive cells were fixed, stained and photographed (c); quantitative results of invading cells (d). Cells were counted under a microscope from ten random fields.

The MCF-7 cells’ migration and invasion towards an extracellular matrix were also investigated under different conditions (Fig. 4a). With a 24 h reaction, the ratio of wound healing was largest (75.6%) in the control group, while in the TiO_2_–Au–TPP group, X-ray group and TiO_2_–Au with X-rays group, the ratios were 71.6%, 64.4% and 49.4%, respectively (Fig. 4b). It is worth noting that the cell marks in the TiO_2_–Au–TPP with X-rays treatment group (25.1%) were wider than those in the other groups, indicating that the nanosensitizer was used under the action of X-rays and could weaken the ability of tumor cells to migrate to their greatest extent. Similar to the results of the migration experiment, the number of invasive cells in the control group and the TiO_2_–Au–TPP-treated cell group was much higher than that of the other groups, indicating that the individual nanosensitizer did not affect the invasive ability of the cells (Fig. 4c and d). The number of invasive cells in the TiO_2_–Au with X-rays group significantly decreased, and that in the TiO_2_–Au–TPP with X-rays group was the lowest under the irradiation of X-rays.

### The therapeutic effect of the nanosensitizer in mice

**Fig. 5:**
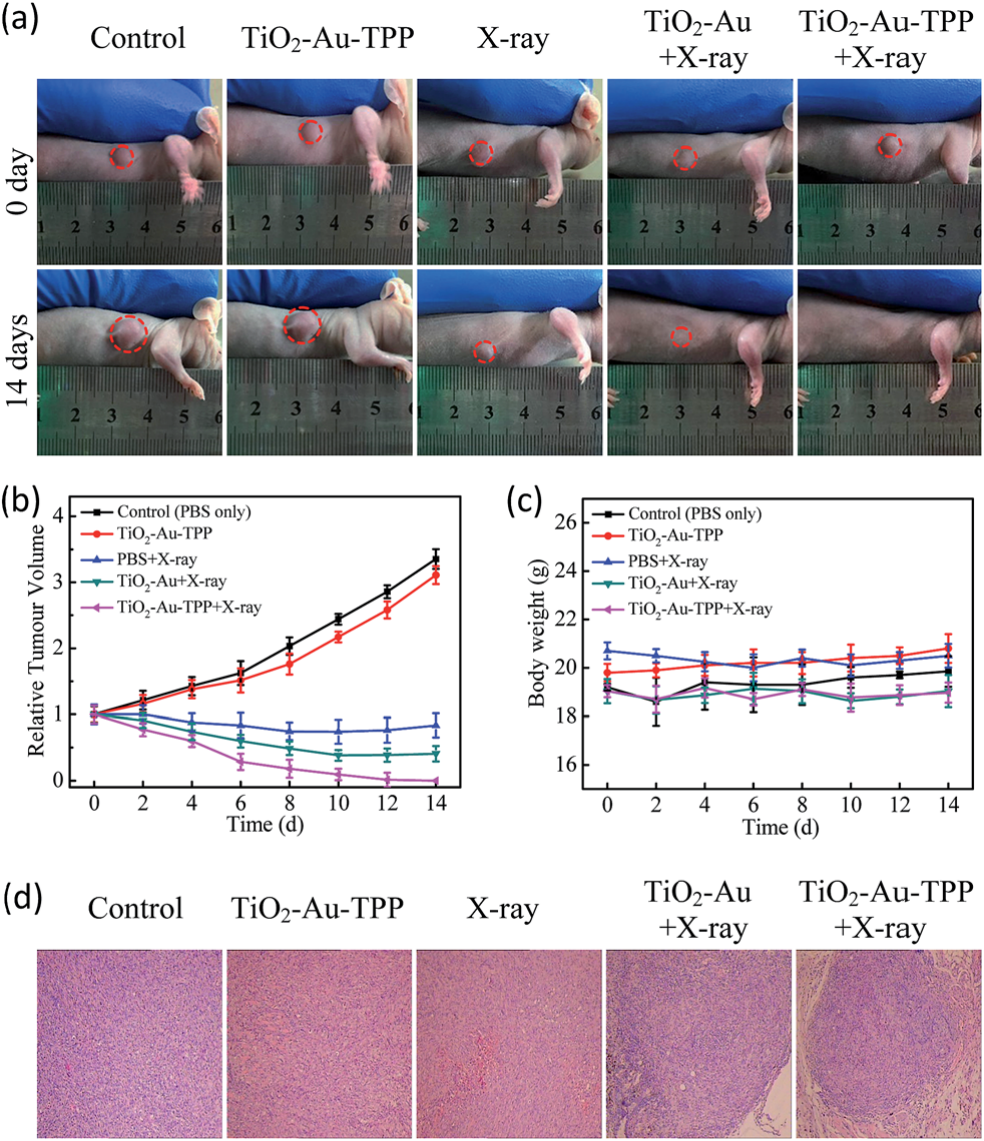
*In vivo* application of the nanoradiosensitizer. Photographs of the mice taken before treatment (0 days) and at 14 days with different treatments (a); tumor growth curves (b) and mouse body weight curves (c) of different groups of tumor-bearing mice. They were measured at a 2 day interval for 14 days; H&E staining of the tumor slides (d) where the tumors were treated differently.

The MCF-7 xenograft tumor-bearing mouse model and 4T1 tumor-bearing Balb/c mouse model were used to evaluate the curing effect of the nanosensitizer on the treatment of tumors *in vivo*. The MCF-7 cells and 4T1 cells (1 × 10^6^ cells per mouse) were first injected into the flanks of nude mice and Balb/c mice, respectively. Nude mice and Balb/c mice were subjected to 5 different treatments, respectively: PBS only, TiO_2_–Au–TPP only, X-rays only, TiO_2_–Au combined with X-ray irradiation and TiO_2_–Au–TPP combined with X-ray irradiation. The nanosensitizer TiO_2_–Au–TPP was dispersed into the PBS buffer solution (1 mg mL^–1^, 50 μL) and intratumorally injected into the mouse tumor, and 6 Gy X-ray irradiation was given 8 h later. As can be seen in Fig. 5a, in the control group treated with PBS buffer, the MCF-7 tumor size was found to increase about 3.5-fold. The MCF-7 tumor volumes of the X-ray group and the TiO_2_–Au–TPP with X-rays group were significantly reduced. However, the MCF-7 xenograft tumor injected with TiO_2_–Au–TPP and irradiated with X-rays completely disappeared after 12 days. In order to evaluate the effect of the nanosensitizer on the treatment of tumors *in vivo*, the Balb/c mice bearing 4T1 murine breast cancer tumor model was also used. As shown in Fig. S11a,† the control groups’ tumor size was found to increase about 7-fold. The 4T1 tumor volumes of the TiO_2_–Au–TPP group, X-ray group and the TiO_2_–Au with X-rays group boosted rapidly for each sample. After 14 days the tumor volumes of the 4T1 tumor stayed the same as that at day 0 when treated with TiO_2_–Au–TPP under 6 Gy of X-ray irradiation. Moreover, since body weight is an essential parameter to evaluate the toxicity to the body, all groups remained almost unchanged over 14 days, which implies that the treatments avoided unpleasant side effects (Fig. 5c and S11c, ESI†). In addition, the survival time of the MCF-7 xenograft tumor-bearing mouse in the TiO_2_–Au–TPP with X-rays treatment group was considerably prolonged, with a 50-day survival rate of 100%, which indicates remarkable therapeutic efficacy and high biocompatibility (Fig. S9, ESI†). The *in vivo* treatment was also evaluated using the H&E staining method: MCF-7 xenograft tumor and 4T1 tumor tissue of the TiO_2_–Au–TPP with X-rays treatment group was subject to widespread damage, while the tumor tissues of the other groups did not change significantly (Fig. 5d and S11d†). This result shows that the nanosensitizer can effectively kill tumor cells, leading to tumor tissue damage. The histological effect of the nanosensitizer on the five major organs (liver, lung, spleen, kidney, and heart) of healthy nude mice was monitored at 7 days after intratumoral injection and no histopathological abnormalities were found (Fig. S10, ESI†). These results illustrate that the nanosensitizer could significantly enhance the therapeutic efficacy of RT.

## Conclusions

In summary, we have demonstrated a novel nanoradiosensitizer initiating a domino effect on mitochondrial ROS burst for RT against cancer, which can not only enhance RT efficacy in living cells and *in vivo*, but also reduce the damage to normal tissues surrounding the tumor targets. The nanoradiosensitizer was constructed with a satellite structure based on TiO_2_ and Au NPs, then linked with TPP to target mitochondria. Confocal laser images demonstrated that the nanosensitizer was able to specifically localize in mitochondria and induce the domino effect on mitochondrial ROS burst for enhancing RT. In addition, the mitochondrial oxygen consumption rate and membrane potential experiments verified that the nanosensitizer could cause an intracellular ROS outbreak in the presence of X-rays, leading to mitochondrial dysfunction and tumor cell death. A cell clone formation assay showed that the cell survival rate of cells with the mitochondrial targeted nanosensitizer was significantly lower than that of other non-targeted groups. Notably, the tumor was significantly suppressed even just once RT with the mitochondria-targeted nanosensitizer. The nanosensitizer in the radiotherapy process reduces the X-ray dose and the number of treatments, shortens the treatment cycle, and effectively lowers the side effects of radiation. The current strategy could provide new perspectives for RT sensitization in future clinical cancer therapy.

## Conflicts of interest

There are no conflicts to declare.

## Supplementary Material

Supplementary information

## References

[cit1] Delaney G., Jacob S., Featherstone C., Barton M. (2005). Cancer.

[cit2] Siva S., Kothari G., Muacevic A., Louie A. V., Slotman B. J., Teh B. S., Simon S. L. (2017). Nat. Rev. Urol..

[cit3] Onufrey V., Mohiuddin M. (1985). Int. J. Radiat. Oncol., Biol., Phys..

[cit4] Lucky S. S., Soo K. C., Zhang Y. (2015). Chem. Rev..

[cit5] Kamkaew A., Chen F., Zhan Y., Majewski R. L., Cai W. (2016). ACS Nano.

[cit6] Chen H., Wang G. D., Chuang Y.-J., Zhen Z., Chen X., Biddinger P., Hao Z., Liu F., Shen B., Pan Z., Xie J. (2015). Nano Lett..

[cit7] Fan W., Shen B., Bu W., Chen F., Zhao K., Zhang S., Zhou L., Peng W., Xiao Q., Xing H., Liu J., Ni D., He Q., Shi J. (2013). J. Am. Chem. Soc..

[cit8] Xiao Q., Zheng X., Bu W., Ge W., Zhang S., Chen F., Xing H., Ren Q., Fan W., Zhao K., Hua Y., Shi J. (2013). J. Am. Chem. Soc..

[cit9] Zhang C., Zhao K., Bu W., Ni D., Liu Y., Feng J., Shi J. (2015). Angew. Chem., Int. Ed..

[cit10] Yu Z., Pan W., Li N., Tang B. (2016). Chem. Sci..

[cit11] Lim S. H., Thivierge C., Nowak-Sliwinska P., Han J., van den Bergh H., Wagnieres G., Burgess K., Lee H. B. (2010). J. Med. Chem..

[cit12] Hatz S., Lambert J. D. C., Ogilby P. R. (2007). Photochem. Photobiol. Sci..

[cit13] Rajaputra P., Nkepang G., Watley R., You Y. (2013). Bioorg. Med. Chem..

[cit14] Balaban R. S., Nemoto S., Finkel T. (2005). Cell.

[cit15] Hoye A. T., Davoren J. E., Wipe P. (2008). Acc. Chem. Res..

[cit16] Kroemer G., Galluzzi L., Brenner C. (2007). Physiol. Rev..

[cit17] Wallace D. C. (1999). Science.

[cit18] Lin M. T., Beal M. F. (2006). Nature.

[cit19] Aon M. A., Cortassa S., Maack C., O’Rourke B. (2007). J. Biol. Chem..

[cit20] Green D. R., Reed J. C. (1998). Science.

[cit21] Aronis A., Melendez J. A., Golan O., Shilo S., Dicter N. (2003). Cell Death Discovery.

[cit22] Yu Z., Sun Q., Pan W., Li N., Tang B. (2015). ACS Nano.

[cit23] Akpan U. G., Hameed B. H. (2010). Appl. Catal., A.

[cit24] Li N., Wang H., Xue M., Chang C., Chen Z., Zhuo L., Tang B. (2012). Chem. Commun..

[cit25] Aon M. A., Cortassa S., O’Rourke B. (2004). Proc. Natl. Acad. Sci. U. S. A..

[cit26] Aon M. A., Cortassa S., Aka F. G., Brown D. A., Zhou L., O’Rourke B. (2009). Int. J. Biochem. Cell Biol..

[cit27] Ly J. D., Grubb D. R., Lawen A. (2003). Apoptosis.

[cit28] Braidot E., Petrussa E., Macrì F., Vianello A. (1998). Biol. Plant..

[cit29] Johnson L. V., Walsh M. L., Chen L. B. (1980). Proc. Natl. Acad. Sci. U. S. A..

[cit30] Lam M., Oleinick N. L., Nieminen A. L. (2001). J. Biol. Chem..

[cit31] Ly J. D., Grubb D. R., Ly J. D., Grubb D. R., Lawen A. (2003). Apoptosis.

[cit32] Dixon S. J., Stockwell B. R. (2014). Nat. Chem. Biol..

[cit33] Rico-Bautista E., Zhu W., Kitada S., Ganapathy S., Lau E., Krajewski S., Ramirez J., Bush J. A., Yuan Z., Wolf D. A. (2013). OncoTargets Ther..

[cit34] Shchepina L. A., Pletjushkina O. Y., Avetisyan A. V., Bakeeva L. E., Fetisova E. K., Izyumov D. S., Saprunova V. B., Vyssokikh M. Y., Chernyak B. V., Skulachev V. P. (2002). Oncogene.

[cit35] Symersky J., Osowski D., Walters D. E., Mueller D. M. (2012). Proc. Natl. Acad. Sci. U. S. A..

[cit36] Brennan J. P., Southworth R., Medina R. A., Davidson S. M., Duchen M. R., Shattock M. J. (2006). Cardiovasc. Res..

[cit37] Li N., Ragheb K., Lawler G., Sturgis J., Rajwa B., Melendez J. A., Robinson J. P. (2003). J. Biol. Chem..

[cit38] Wen L., Chen L., Zheng S., Zeng J., Duan G., Wang Y., Wang G., Chai Z., Li Z., Gao M. (2016). Adv. Mater..

