# Supplementary material for: A mitochondria-targeted nanoradiosensitizer activating reactive oxygen species burst for enhanced radiation therapy
Source: Chem Sci. 2018 Feb 28;9(12):3159–64. doi: 10.1039/c7sc04458e (PMC5916111; doi:10.1039/c7sc04458e)
Supplement: Supplementary file 1 [file SC-009-C7SC04458E-s001.pdf]

Electronic Supplementary Material (ESI) for Chemical Science.
This journal is © The Royal Society of Chemistry 2018

Supplementary Information

A mitochondria-targeted nanoradiosensitizer activating reactive oxygen species burst for enhanced radiation therapy

Na Li,[a] Longhai Yu,[a] Jianbo Wang,[b] Xiaonan Gao,[a] Yuanyuan Chen,[a] Wei Pan[a] and Bo Tang[a]*

[a]College of Chemistry, Chemical Engineering and Materials Science, Collaborative Innovation Center of Functionalized Probes for Chemical Imaging in Universities of Shandong, Key Laboratory of Molecular and Nano Probes, Ministry of Education, Institute of Molecular and Nano Science, Shandong Normal University, Jinan 250014, P. R. China.

[b]Radiation Department, Qilu Hospital of Shandong University, Jinan 250100, P. R. China.

E-mail: tangb@sdnu.edu.cn

Experimental Section:

Materials. Tetrabutyl titanate (TBOT), dimethyl sulfoxide (DMSO), glacial acetic acid, methanol, hydrogen tetrachloraurate (III) ($HAuCl_4 \cdot 4H_2O$) and ethanol were purchased from China National Pharmaceutical (Shanghai, China); 2-(N-morpholino)ethanesulfonic acid (MES), (3-aminopropyl)triethoxysilane (APTES), (4-carboxybutyl)triphenylphosphonium (TPP), 1-ethyl-3-(3-dimethlyaminopropyl) carbodiimide (EDC), N-hydroxysuccinimide (NHS) and 4-(hydroxymethyl)phosphonium chloride solution 80% in H_2O (THPC) were obtained from Alfa Aesar (Tianjin, China); hydroethidine (HE), IR806, oligomycin, carbonyl cyanide 4-(trifluoromethoxy)phenylhydrazone (FCCP) and rotenone were purchased from Sigma; Mito-Tracker Green (MTG) was purchased from Molecular Probes (Invitrogen, U.S.); cyclosporine A (CsA) and N-(2-hydroxyethyl) piperazine-N'-(2-ethanesulfonic) acid (HEPES) was obtained from Sangon (Shanghai, China); anti-caspase-3 antibody was purchased from Boster (Wuhan, China.); fluorescein (FITC)–conjugated affinipure goat anti-rabbit IgG (H + L) was purchased from Proteintech (Chicago, U.S.); the human breast cancer cell line (MCF-7) was purchased from KeyGEN biotechnology (Nanjing, China); analytical grade reagents were used with no further purification. All aqueous solutions were prepared using distilled-deionized water of 18.2 MΩ·cm.

Instruments. High resolution transmission electron microscopy (HRTEM) was carried out on a JEM-2100 electron microscope. The crystal structure of the samples were determined by powder X-ray diffraction (PXRD) patterns (Bruker D8, Germany). Fluorescence spectra were acquired with fluorescence spectrometer (FLS-980, Edinburgh, UK). UV-Vis absorption spectra were measured on a pharmaspec UV-1700 UV-Visible spectrophotometer (Shimadzu, Japan). All pH measurements were performed with a pH-3c digital pH-meter (Shanghai LeiCi, China). Confocal fluorescence imaging studies were performed with a TCS SP5 confocal laser scanning microscopy (Leica, Germany). Oxygen consumption rate was measured in an XF24 Extracellular Flux Analyzer (Seahorse XFe24, U.S.). X-ray radiotherapy at a power of 6 MV for 4 Gy or 6 Gy, the dose rate of the irradiation was 300 cGy/min and the source skin distance = 100 cm (Siemens Primus HI, Germany).

Preparation of titanium dioxide nanoparticles (TiO_2 NPs). TiO_2 NPs were synthesized according to a previously reported sol-gel method.[1] Typically, 5 mL TBOT was added to 20 mL ethanol and the mixture was stirred for 10 min to obtain solution A. Meanwhile, 6 mL glacial acetic acid and 1.5 mL water was added to 20 mL absolute ethanol to make solution B. Afterwards, solution A was added dropwisely to solution B and then the reaction was stirred for 30 min before heating to 60 °C for 24 h. The resultant colloidal solution turns to a clear and transparent sol, which was further aged at 80 °C for 24 h to yield a yellowish solid product. Finally, the product was annealed at 400 °C for 3 h to remove the impurities.

Preparation of amino-functionalized TiO_2 NPs (TiO_2-NH_2). TiO_2 NPs' surface was functionalized with amino groups by treatment with APTES.[2] As-prepared TiO_2 NPs (4 mg) was dispersed in a mixed solution of ethanol (20 mL) and water (200 μL) under stirring for 15 min. Then, 20 μL APTES (80 μmol) was added to the mixture and stained for 12 h. After centrifugation (10,000 rpm/min, 10 min) and three times of washing with water, amino-functionalized TiO_2 NPs were redispersed in 8 mL of MES buffer (20 mM, pH = 6.0).

Preparation of gold nanoparticles. The Au NPs were prepared by reduction of $HAuCl_4$ solution *via* THPC. 6 μL THPC (80% solution in water) was added to 23.25 mL NaOH solution (10 mM) and the mixture was stirred for 10 min. Followed by a rapid addition of $HAuCl_4 \cdot 4H_2O$ solution (0.75 mL, 1% by mass) and stirring at room temperature for 15 min, a yellowish Au NPs solution was obtained. The solution was stored at 4 °C for later use.

Preparation of TiO_2-Au NPs. Au NPs were anchored on the surface of TiO_2-NH_2 by the coordination bonds. 4 mg as-prepared TiO_2-NH_2 NPs were added to 24 mL yellowish Au NPs solution. The mixture was then stirred at room temperature for 24 h. Finally, the precipitates were centrifuged (10,000 rpm/min, 10 min), washed with water and PBS buffer (10 mM, pH = 7.4) for three times, and redispersed in PBS buffer (1 mg/mL).

Preparation of TiO_2-Au-TPP and TiO_2-Au-TPP-IR806. TiO_2-Au-TPP-IR806 was prepared by coupling the carboxyl groups of the TPP bromide and infrared dye IR806 with the amino group on the surface of TiO_2-Au to form the amido bonds. EDC, NHS, IR806 and TPP bromide was added in 10 mL of MES buffer (10 mM, pH = 6.0) and amount of EDC, NHS, IR806 and TPP bromide are shown in Table 1. The reaction was performed for 1 h at room temperature in the dark to activate carboxylate groups and then both of them were added to above TiO_2-Au solution under gentle stirring for 24 h, which lead the amido bonds formation. Later, the precipitates were centrifuged (10,000 rpm/min, 10 min), and washed with methanol and PBS buffer (10 mM, pH = 7.4) for three times, and finally redispersed in PBS buffer (1 mg/mL). The content of TPP bromide groups were calculated according to the standard linear calibration curve of each group using subtraction through UV-Vis absorption spectra.

Table 1. The amount of EDC, NHS, IR806 and TPP bromide.

EDC (μmol)	NHS (μmol)	IR806 (μmol)	TPP bromide (μmol)
80	80	2	8
120	120	2	12
160	160	2	16
200	200	2	20
240	240	2	24

Preparation of TiO_2-Au-TPP-HE. As-prepared TiO_2-Au and TiO_2-Au-TPP were further modified with carboxyl groups.[3] 4 mg TiO_2-Au-TPP were dispersed in 8 mL DMSO containing triethylamine (2 mg) and succinic anhydride (2 mg). The mixture was allowed to stir at 40 °C for 48 h and then centrifuged (10,000 rpm/min, 10 min) to collect the NPs. The NPs were washed three times with water and finally redispersed in MES buffer (20 mM, pH = 6.0). HE molecule was anchored on the TiO_2-Au-TPP by formation of amido bonds. EDC (200 μmol)

and NHS (200 μmol) were added to the carboxylic acid-functionalized NPs with reaction for 1 h to activate carboxylate groups and then HE molecule (20 μmol) in methanol was added to solution under gently stirring for 24 h in the dark, which resulted in the formation of amido bonds. The precipitates were centrifuged (10 000 rpm/min, 10 min) and washed with deionized water and PBS buffer (10 mM, pH = 7.4) for three times and finally redispersed in PBS buffer (1 mg/mL).

Standard linear calibration curve of $O_2^{\bullet-}$. According to previous report, xanthine oxidase can catalyze 1.00×10^{-6} mol xanthine into 0.33×10^{-6} mol $O_2^{\bullet-}$ per unit.[4] Xanthine (0.03, 0.06, 0.08, 0.12, 0.15, 0.18 mmol), xanthine oxidase (0.03, 0.06, 0.08, 0.12, 0.15, 0.18 U) and hydroethidine (0.02 μmol) were added to 2 mL HEPES buffer (0.02 M, pH = 7.4), respectively. The mixture was maintained at 37 °C for 30 min. The fluorescence spectra of hydroethidine were obtained by excitation of the samples at 488 nm (emission = 520-620 nm).

Cell culture. MCF-7 cells were cultured in Dulbecco's modified Eagles medium (DMEM). All cells were supplemented with 10% fetal bovine serum and 100 U/mL of 1% antibiotics penicillin/streptomycin to maintain at 37 °C in a 100% humidified atmosphere containing 5% CO_2.

Triphenylphosphine (TPP) concentration optimization. MCF-7 cells were cultured in a confocal dish and incubated at 37 °C in 5% CO_2 in DMEM for 24 h. Then, A series of nanoparticles (TiO_2-Au-TPP-IR806, 0.1 mg/mL) with different concentrations of TPP (the TPP loading capacity to 8, 12, 16, 20, 24 μmol) were delivered into the cells in DMEM for 8 h. The cells were then washed three times with PBS buffer (10 mM, pH = 7.4) to remove the NPs that were not taken up into the cells. Next, 1 mL fresh DMEM was added and the cells were stained by Mito-Tracker Green (20 nM) at 37 °C for 20 min. The cells were then washed by PBS buffer (10 mM, pH = 7.4) several times and observed using CLSM with 488 nm excitation for Mito-Tracker Green (emission = 500-550 nm) and 633 nm excitation for IR806 (emission = 750-800 nm). The colocalization effect of Mito-Tracker Green with IR806 of TiO_2-Au-TPP-IR806 was quantified using Image-Pro Plus Image analysis software.

***In vitro* detecting $O_2^{\bullet-}$ burst.** Real-time monitoring $O_2^{\bullet-}$ experiment was carried out. MCF-7 cells were plated in a confocal dish for 24 h and then TiO_2-Au-TPP-HE (0.1 mg/mL) was delivered into the cells in DMEM for 8 h at 37 °C in 5% CO_2. The cells were washed three

times with PBS buffer (10 mM, pH = 7.4) to remove the out cell excess NPs. After that, 2 mL fresh DMEM containing 10% fetal bovine serum medium was added and followed with irradiation with X-ray for 4 Gy. Afterwards, MCF-7 cells were imaged through CLSM with 488 nm excitation for 12 h (emission = 500-550 nm) with 2 h interval.

Detection of mitochondrial membrane potential ($\Delta\Psi_m$). MCF-7 cells were plated in a confocal dish for 24 h. Then, TiO_2-Au-TPP (0.1 mg/mL) was then delivered into the cells in DMEM culture medium for 8 h and then the cells were washed three times with PBS buffer (10 mM, pH = 7.4). Then the cells were irradiated for 4 Gy and further incubated for 4 h. Afterwards, 1 mL fresh DMEM culture medium was added and the cells were incubated with Rhodamine 123 (5 μg/mL) at 37 °C for 15 min under dark. CLSM of Rhodamine 123-stained cells were imaged by excitation of the samples at 488 nm (emission = 500-550 nm).

Measurement of oxygen consumption. Oxygen consumption rate (OCR) was measured in real-time, in an XF24 Extracellular Flux Analyzer.[5] MCF-7 cells were seeded in XF24-well plates (7000 cells per well in 100 μL culture medium). After 6 h, 200 μL media was added and followed by incubation overnight at 37 °C under 5% CO_2. The cells were subjected to 3 different treatments: group 1 with X-ray only, group 2 with TiO_2-Au and X-ray irradiation and group 3 with TiO_2-Au-TPP and X-ray irradiation. After 8 h, X-ray treatment was performed with X-ray for 4 Gy and medium was replaced with serum and bicarbonate-free assay medium 1 h before oxygen consumption rate. After incubation, the sensor cartridge was loaded with oligomycin (10 μM, port A), carbonyl cyanide 4-(trifluoromethoxy) phenylhydrazone (FCCP, 20 μM, port B) and rotenone (10 μM, port C) to measure the OCR. Cells were washed once with serum-free un-buffered assay medium containing 10 mM sodium pyruvate and 25 mM glucose. Before OCR measurement, cells were pre-incubated with nanoprobes in regular media for 8 h followed by washing once with assay medium, and cells were kept in 500 μL/well of assay medium. Once the sensor cartridge was equilibrated, the calibration plate was replaced with the assay plate.

Colony formation assay. MCF-7 cells were cultured in 60 mm dishes and incubated at 37 °C under 5% CO_2 in DMEM for 24 h. The cells were then subjected to 5 different treatments: blank as the control group, TiO_2-Au-TPP only, X-ray irradiation only, TiO_2-Au with X-ray irradiation and TiO_2-Au-TPP with X-ray irradiation. After 8 h, the cells were washed three times with PBS

buffer (10 mM, pH = 7.4) to remove the excess NPs that were not uptaken into the cells. Subsequently, X-ray treatment was performed with X-ray for 4 Gy and those cells were then incubated in fresh cell culture medium at 37 °C under 5% CO_2 in DMEM for another 10 days, before they were fixed with 4% paraformaldehyde and stained with 0.2% crystal violet. The resulting colonies with more than 50 cells each were counted. The surviving fraction = (surviving colonies) / (cells seeded × plating efficiency). The mean surviving fraction was obtained from three parallel tests.[6]

Cell migration assay. The same setup of colony formation assay was applied with the Migration assay until washing with PBS buffer for three times and then the X-ray treatment. The cells were wounded by dragging a 10 µL pipette tip through the monolayer. Followed by three times washing with PBS, cellular debris was removed and images were acquired at the time of 0, 12 and 24 h post-wounding.

Cell invasion assay. For invasion assay, the same setup was again applied until the X-ray irradiation. 2×10^4 trypsinized cells were added into the upper compartment and further incubated for 24 h. The noninvasive cells on the upper surface of the membrane were removed by a cotton-tipped swab. The invasive cells which adhered to the lower surface of the membrane were then fixed with 4% paraformaldehyde and stained with 0.2% crystal violet before counting the number of invaded cells under microscope.

Caspase-3 activation. MCF-7 cells were incubated with 0.1 mg/mL of TiO_2-Au-TPP at 37 °C in 5% CO_2 for 8 h. The cells were then washed three times with PBS buffer (10 mM, pH = 7.4) to remove the nanosensitizer. After that, 1 mL fresh DMEM containing 10% fetal bovine serum medium was added and followed with irradiation with X-ray for 4 Gy. After further incubation for 12 h, the cells were fixed with paraformaldehyde (4%) for 15 min, and then treated with primary antibody anti-caspase-3 (10 µg/mL), enhanced secondary antibody for 1 h and 45 min at 37 °C, respectively. At last, the cells were washed with PBS buffer for several times and observed through CLSM with 488 nm excitation for FITC (emission = 500-550 nm).

Animal tumor models. Female nude mice and Balb/c mice (4-6 weeks old, ~20 g) were housed under normal conditions with 12 h light and dark cycles and given access to food and water ad libitum. MCF-7 cells and 4T1 cells were suspended and harvested after trypsinization and approximately 1×10^6 cells in 200 µL PBS buffer (10 mM, pH = 7.4) were injected

subcutaneously into the flank of the nude mice and Balb/c mice. The tumor volume (V) was determined by measuring length (L) and width (W), and calculated as $V = L \times W^2 / 2$. The relative tumor volumes were calculated for each mouse as V / V_0 (V_0 was the tumor volume when the treatment was initiated).

***In vivo* antitumor efficacy *via* injection.** When the tumor volume reached to about 80-100 mm^3, the tumor-bearing mice were weighed and randomly divided into different groups ($n \geq 5$). The mice were subjected to 5 different treatments: PBS only, TiO_2-Au-TPP only, X-ray only, TiO_2-Au combined with X-ray irradiation and TiO_2-Au-TPP combined with X-ray irradiation. 50 μL PBS, 50 μL 1.0 mg/mL TiO_2-Au-TPP or TiO_2-Au in PBS were intratumorly injected into the corresponding group. After 8 h, X-ray treatment was performed on group 2, 3, and 4 by irradiating the tumor region with X-ray for 6 Gy. The tumor sizes and the body weights of the mice were measured every other day for 14 days. The survival outcomes of the mice were determined by tumor volumes greater than 1000 mm^3 or clinically mandated euthanasia. Haematoxylin and eosin (H&E) staining was carried out at 12 h post-treatment for the tumors and at 7 days post-treatment for the five major organs.

References

1 U. G. Akpan and B. H. Hameed, *Appl. Catal., A*, 2010, **375**, 1-11.

2 F. Muhammad, M. Guo, W. Qi, F. Sun, A. Wang, Y. Guo and G. Zhu, *J. Am. Chem. Soc.*, 2011, **133**, 8778-8781.

3 X. Gao, C. Ding, A. Zhu and Y. Tian, *Anal. Chem.*, 2014, **86**, 7071-7078.

4 J. Hu, N.-K. Wong, S. Ye, X. Chen, M.-Y. Lu, A. Q. Zhao, Y. Guo, A. C.-H. Ma, A. Y.-H. Leung, J. Shen and D. Yang, *J. Am. Chem. Soc.*, 20215, **137**, 6837-6843.

5 E. Rico-Bautista, W. Zhu, S. Kitada, S. Ganapathy, E. Lau, S. Krajewski, J. Ramirez, J. A. Bush, Z. Yuan and D. A. Wolf, *Onco Targets Ther.*, 2013, **4**, 1212-1229.

6 L. Wen, L. Chen, S. Zheng, J. Zeng, G. Duan, Y. Wang, G. Wang, Z. Chai, Z. Li and M. Gao, *Adv. Mater.*, 2016, **28**, 5072-5079.

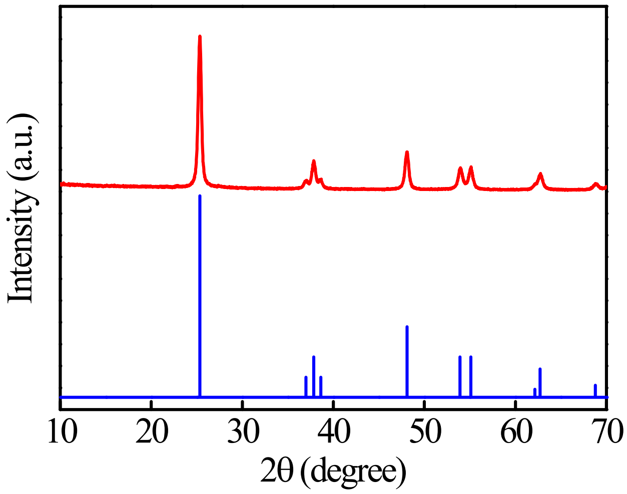

Fig. S1 The PXRD spectrum of TiO_2 NPs. The ICDD-PDF overlay of anatase TiO_2 (PDF No. 21-1272) is shown as vertical line.

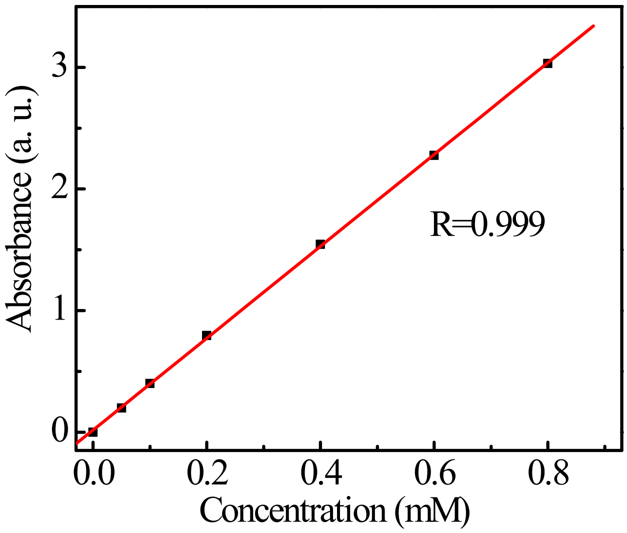

Fig. S2 Standard linear calibration curve of TPP.

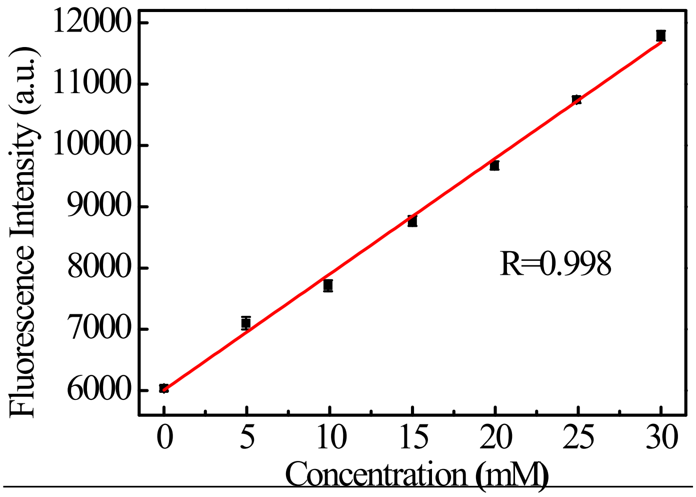

Fig. S3 Standard linear calibration curve of $O_2^{\bullet-}$.

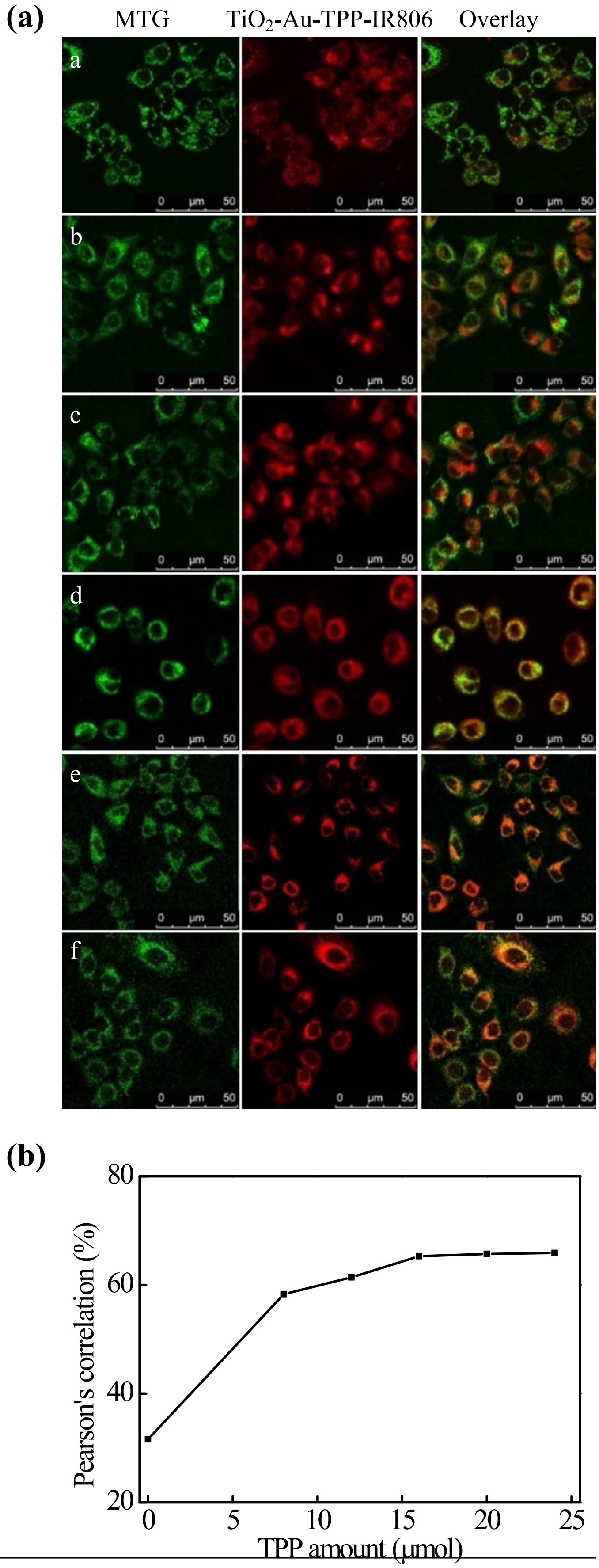
(a)
MTG
TiO2-Au-TPP-IR806
Overlay
a
b
c
d
e
f
0 µm 50
(b)
Pearson's correlation (%)
TPP amount (µmol)
80
60
40
20
0
5
10
15
20
25

Fig. S4 Mitochondrial targeting of TiO_2-Au-TPP-IR806 with different concentrations of TPP groups under confocal imaging. (a) Confocal images of TiO_2-Au-TPP with different the amount of TPP was added (a-f: 0, 8, 12, 16, 20, 24 μmol); (b) the colocalization effect of TiO_2-Au-TPP-IR806 with different the amount of TPP was added.

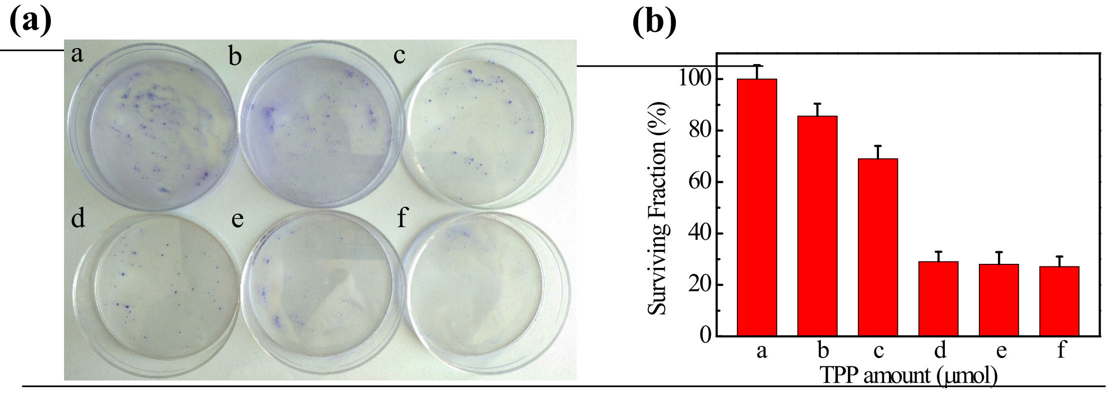

Fig. S5 (a) Photograph of MCF-7 cells surviving fractions after incubated TiO_2-Au-TPP with different the amount of TPP loading (a-f: 0, 8, 12, 16, 20, 24 μmol); (b) corresponding surviving fractions of MCF-7 cells for (a).

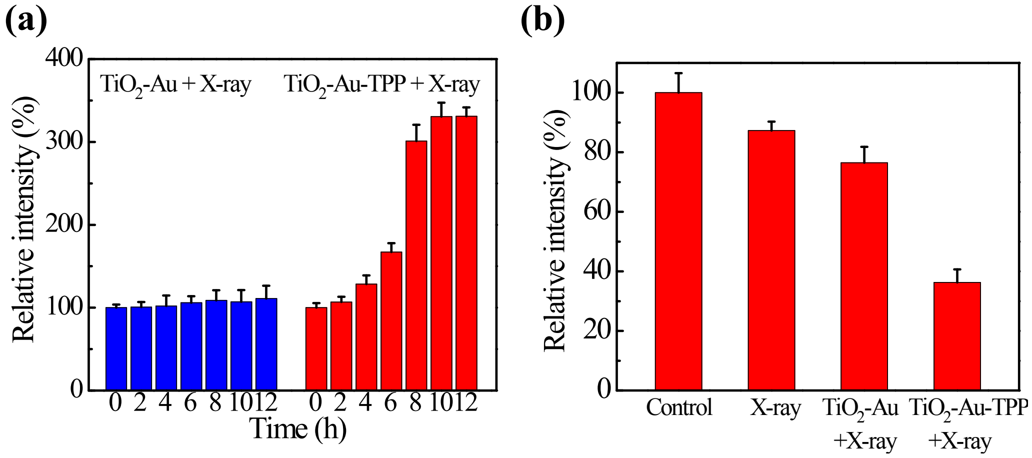

Fig. S6 (a) Relative fluorescence intensity of MCF-7 cell incubated with TiO_2-Au-HE or TiO_2-Au-TPP-HE at 12 h post irradiation; (b) relative fluorescence intensity of Rhodamine 123-stained cells with different treatment.

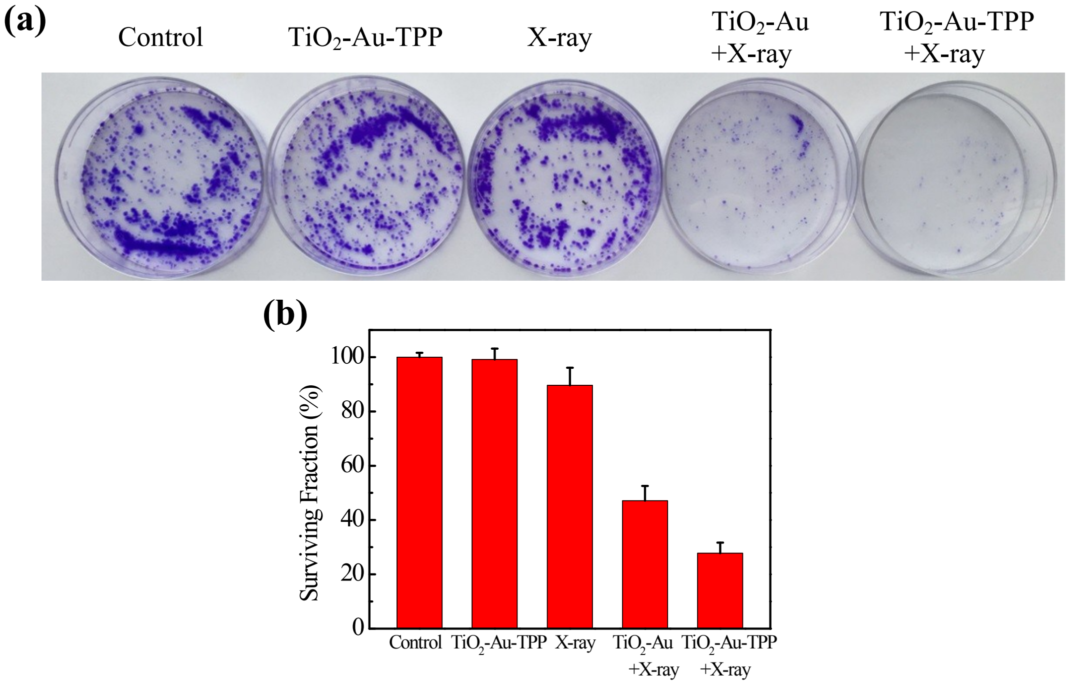

Fig. S7 Cell clone formation assay of MCF-7 cells treated differently and incubated in the presence of CsA (1 μM). (a) Photograph of MCF-7 cells surviving fractions after the different treatments; (b) surviving fractions of MCF-7 cells.

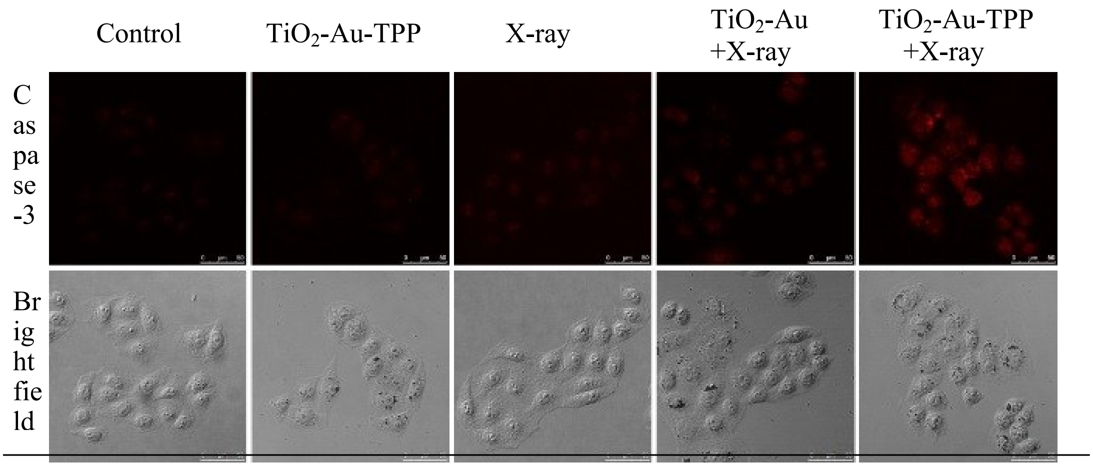

Fig. S8 Immunofluorescent staining images of caspase-3 after the different treatments.

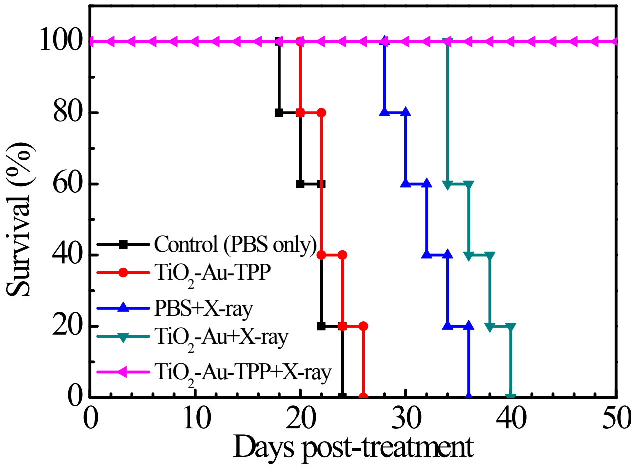

Fig. S9 Survival rates for each group after receiving different treatments.

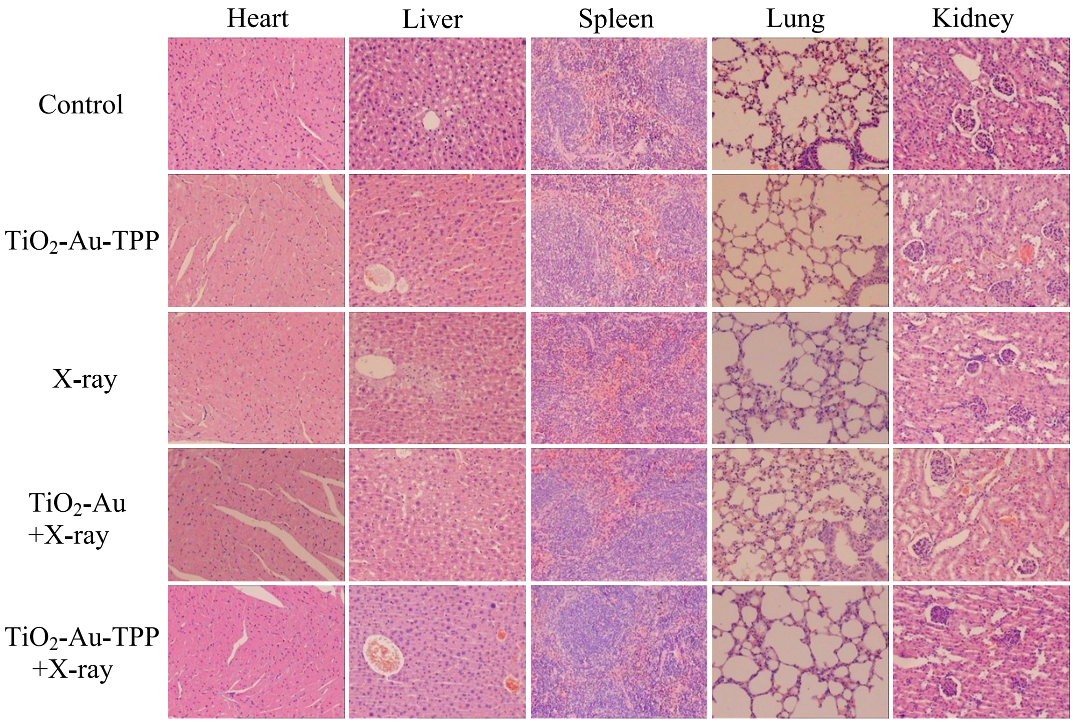

Fig. S10 Biocompatibility of the nanoparticles. H&E staining images of five major organs (heart, liver, spleen, lung, and kidney) at 7 day after different treatment groups: PBS only; TiO_2-Au-TPP only; X-ray only; TiO_2-Au with X-ray; TiO_2-Au-TPP with X-ray and no histopathological abnormalities were found in all groups. A dosage of nanoparticles in PBS (1 mg/mL, 50 μL) was administrated intratumorally for all mice.

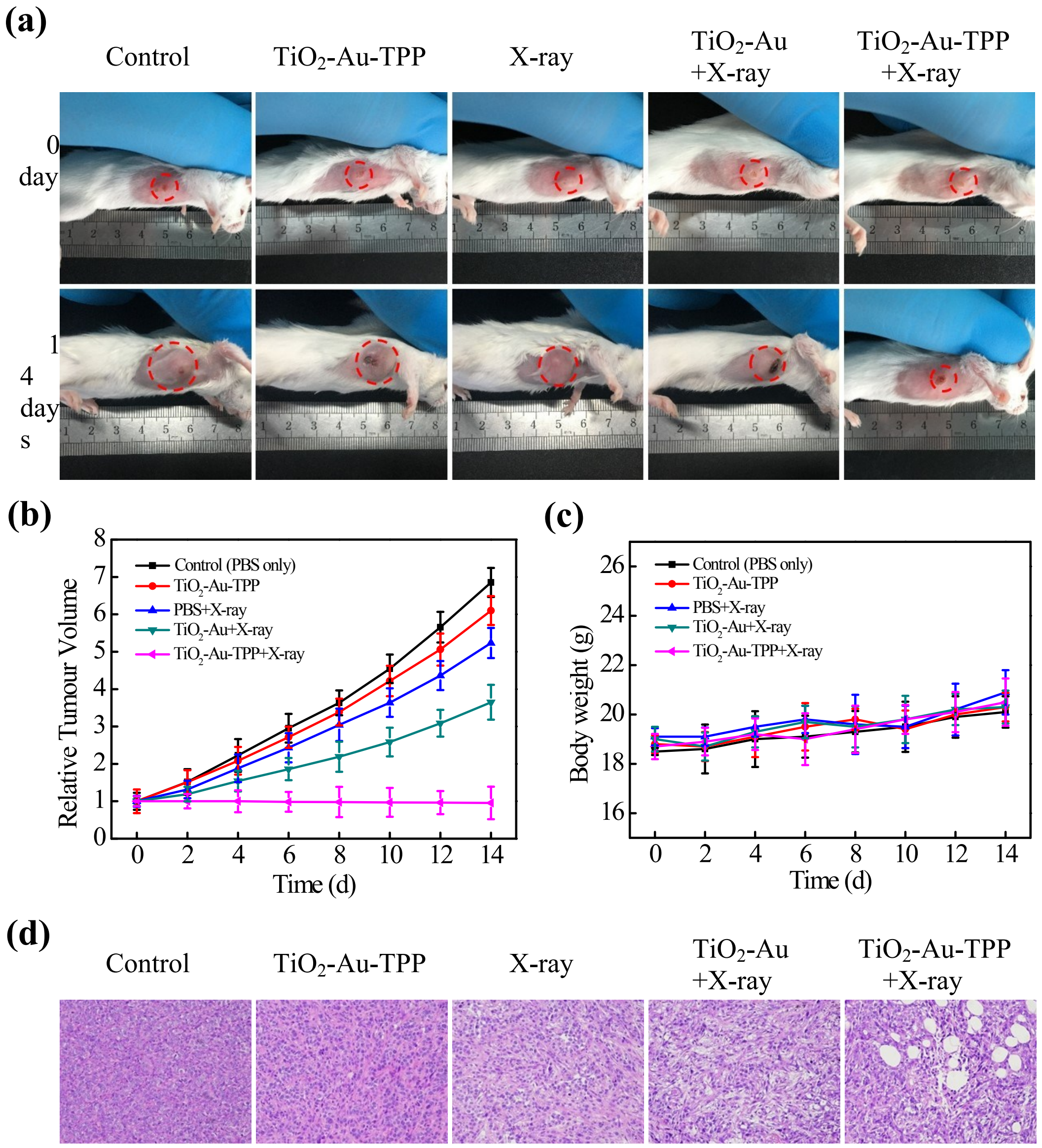

Fig. S11 *In vivo* application of nanoradiosensitizer. (a) Photographs of the mice taken before treatment (0 day) and at 14 days with different treatments; (b) tumor growth curves and (c) mice body weight curves of different tumor-bearing mice groups. All the measurements were taken at 2 days interval for 14 days; (d) H&E staining of tumor slides for corresponding groups.
